# Zero ischemia robotic partial nephrectomy: Oncological and functional outcomes of a multicenter study

**DOI:** 10.55730/1300-0144.5658

**Published:** 2023-02-28

**Authors:** Kemal ENER, Abdullah Erdem CANDA, Murat BİNBAY, Mevlana Derya BALBAY, Ali Fuat ATMACA

**Affiliations:** 1 Department of Urology, Ümraniye Training and Research Hospital, University of Health Sciences, İstanbul, Turkiye; 2 Department of Urology, School of Medicine, Koç University, İstanbul, Turkiye; 3 Department of Urology, School of Medicine, Altınbaş University, İstanbul, Turkiye; 4 Department of Urology, School of Medicine, Ankara Yıldırım Beyazıt University, Ankara, Turkiye

**Keywords:** Kidney, partial nephrectomy, renal cell carcinoma, robotic surgery, zero ischemia

## Abstract

**Background/aim:**

The functional and oncological outcomes of zero ischemia robotic partial nephrectomy (RPN) procedures were evaluated.

**Materials and methods:**

A total of 56 patients underwent zero ischemia RPN transperitoneally, and their data were collected prospectively. Radius, exo/endophytic, nearness, anterior/posterior, location (R.E.N.A.L.) nephrometry, and PADUA scores were calculated. Patient and tumor characteristics were evaluated. Intra- and perioperative (0–30 days) complications were evaluated by Clavien classification. The change in serum creatinine, and estimated glomerular filtration rates (eGFR) were evaluated during preoperative, immediate postoperative periods, and at postoperative 6^th^ months.

**Results:**

The mean age of the patients was 52.2 ± 8.1 (27–75) years. R.E.N.A.L. nephrometry and PADUA scores were 6.1 ± 1.3 and 7.3 ± 1.0, respectively. The duration of surgery was 108.4 ± 18.2 min and estimated blood loss was 166.2 ± 124.7 mL. There were no intraoperative complications in any of the patients. Clavien Grade 1 and 3 complications were seen in 2 patients in the perioperative period. In the perioperative period (1–30 days), one patient required blood transfusion and angiographic intervention due to postoperative bleeding (Clavien Grade 3), and one patient required hospitalisation due to prolonged subileus (Clavien Grade 1) that resolved conservatively. The radiological and pathological tumor sizes were 3.1 ± 1.1 cm and 2.8 ± 1.4 cm, respectively. The surgical margins were positive in two patients with tumour sizes of 1.5 and 4 cm. Neither local recurrence nor distant metastasis was detected, during 33.6 ± 12.3 (3–76) months. There were no statistically significant differences between preoperative eGFR and serum creatinine levels, compared with those of immediate postoperative and postoperative 6^th^ month periods.

**Conclusion:**

Zero ischemia RPN is a safe and applicable method with acceptable oncological and functional outcomes in small renal tumors and even in selected larger renal tumors.

## 1. Introduction

Kidney neoplasms account for approximately 3% of all malignancies in adulthood. Currently, the widespread use of radiological imaging modalities led to the detection of incidental renal tumors, in patients with no symptoms [1]. Today, partial nephrectomy (PN) is preferred in kidney masses <4 cm, and in tumors between 4–7 cm, if suitable [2]. Previously, it has been reported that PN had similar oncologic outcomes with favorable functional outcomes compared with radical nephrectomy (RN) [3]. During robotic and laparoscopic procedures, the failure of kidney cooling is still remaining as a challenging point [4]. The era of da Vinci’s surgical robotic system (Intuitive Surgical Sunnyvale, CA, USA) in renal tumors, has favoured PN during the surgical steps [5].

The on-clamp approach is performed by placing a temporary renal pedicle clamp, which reduces bleeding during tumor resection and provides a better visualization. However, in the literature there are studies, suggesting that ischemia may deteriorate renal functions postoperatively [6]. Therefore, zero ischemia approach is proposed to maintain a better functional outcome, which is especially important in patients that have underlying chronic renal diseases or a solitary kidney. In the current paper, we evaluated the oncological and functional outcomes of zero ischemia RPN procedures performed in three centers by including 56 consecutive patients.

## 2. Materials and methods

This study was approved by the local Institutional Review Board and the consent of each patient for the use of their information was obtained in writing. Between 2013 and 2021, zero ischemia RPN was performed in 56 patients transperitoneally, using the da Vinci robotic surgical system, and their data were collected prospectively. Patients, older than 18 years of age and capable to consent, having an organ-confined renal tumor, and scheduled for elective partial nephrectomy for renal neoplasms were included in the study. Patients who were not feasible for robotic surgery were excluded from the study. Additionally, unwillingness to participate in the study was accepted as an exclusion criterion. In order to determine the anatomical features of the kidney masses, abdominopelvic computed tomography (CT) or magnetic resonance imaging (MRI) was done, and the R.E.N.A.L. nephrometry, and PADUA scores were determined [7,8]. The Clavien-Dindo classification was used to evaluate surgical complications [9]. The Charlson Comorbidity Index (CCI) was used for quantifying the prognosis of patients. The anatomical and histopathological features of the tumors’ and the patient characteristics were evaluated. The serum creatinine levels and estimated glomerular filtration rates (eGFR) were evaluated during preoperative, immediate postoperative periods, and at postoperative 6^th^ months and compared. The eGFRs of the patients were calculated using the Modification of Diet in Renal Disease (MDRD) formula.

### 2.1. Surgical technique

**Figure 1:**
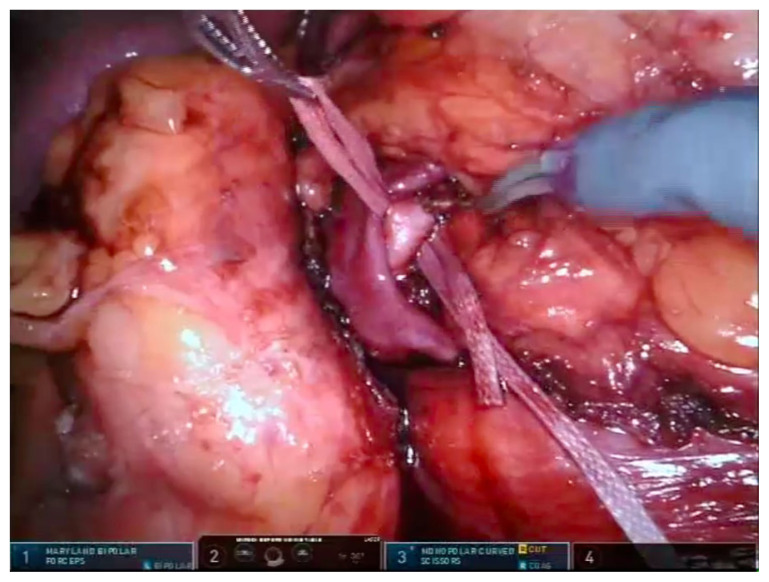
Renal artery is secured with a vascular tape (Dr. Canda’s surgical archive).

**Figure 2:**
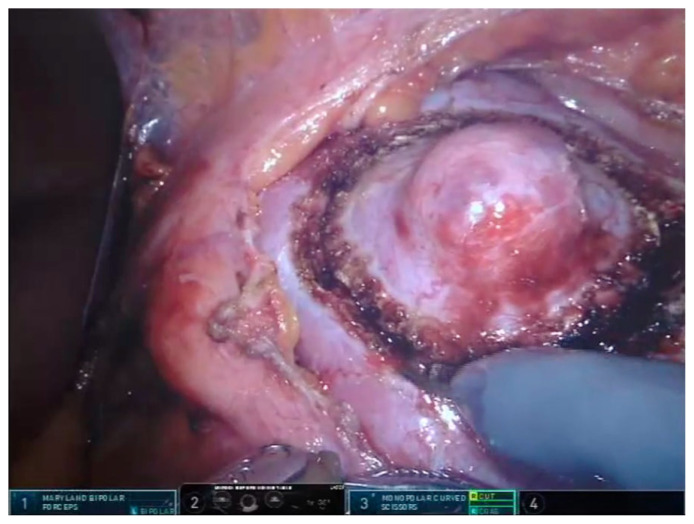
Performing robotic zero ischemia partial nephrectomy with inclusion of few millimetres of normal renal tissue around the tumor (Dr. Canda’s surgical archive).

**Figure 3:**
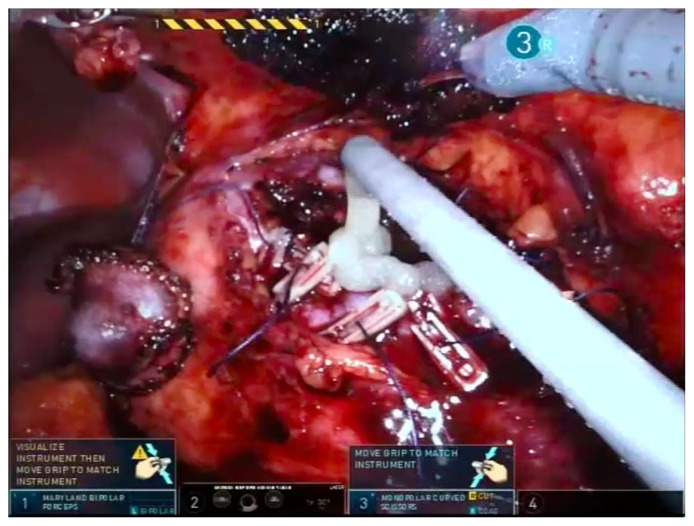
Appearance of the completed robotic zero ischemia partial nephrectomy (Dr. Canda’s surgical archive).

After colon mobilization, the kidney and renal mass is visualized. Hilar dissection is performed in all cases including the zero ischemia procedures. After dissection of renal artery, it is encircled with a vascular tape (Figure 1). In peripheral and exophytic masses, zero ischemia PN is preferred. The tumor is dissected by using the robotic scissors with a tumor free parenchymal margin. Then the mass is placed in an endobag with the adipose tissue on it (Figure 2). For internal and external renorrhaphies, 4-0 V-Loc 90, 1200 30 cm ½ 17 mm (Covidien, New Haven, CT, USA) and 3-0 V-Loc 180, 1800 45 cm, 1/2 26 mm (Covidien) sutures are used respectively. Thereafter, absorbable clips (Lapra-Ty (Ethicon Endosurgery, Inc., Cincinnati, OH, USA)) are placed reciprocally across the sutures (Figure 3). Before the placement of a foley drain, the hemostasis is checked by decreasing the intraabdominal pressure.

### 2.2. Statistical analysis

Statistical analyses were performed using Statistical Package for the Social Sciences (SPSS Inc; Chicago, IL, USA) version 20. The minimum and maximum values of the mean and the standard deviation were used in summarizing the numeric parameters. The Kolmogorov-Smirnov test was used to determine the distribution of the variables. Wilcoxon’s signed-rank test was used as a nonparametric statistical method to compare parameters with a skewed distribution. The level of statistical significance was determined as p = 0.05.

## 3. Results

For eGFR, when the alpha is 0.05 and the sample size is 56, the post hoc power of the study is calculated as 0.84, with the f-value 0.127 in the presence of a single group and 3 measurement times. However, for creatinine, when the alpha is 0.05 and the sample size is 56, the post hoc power of the study is calculated as 0.14, with the f-value 0.041 in the presence of a single group and 3 measurement times.

Forty-one male and 15 female patients were included in the study. The mean age of patients was 52.2 ± 8.1 (27–75) years, and the mean body mass index (BMI) was 27.8 ± 3.5 kg/m^2^. R.E.N.A.L. nephrometry and PADUA scores were 6.1 ± 1.3 and 7.3±1.0, respectively. Of the renal masses, 36 were located on the anterior surface, 14 were located on the posterior surface and 6 were located on the lateral aspects of the kidneys. The mean CCI and ASA scores were 1.3 ± 1.2 and 1.7 ± 0.7, respectively. The mean operating time was 108.4 ± 18.2 min. The mean hemorrhage was 166.2 ± 124.7 mL. No intraoperative complications were seen in the patients. In the perioperative period, blood transfusion and angiographic intervention were required in one patient due to major hemorrhage (Clavien Grade 3). Additionally, one patient was hospitalized, and received pharmacological treatment, because of prolonged subileus (Clavien Grade 1). The mean duration of hospitalization was 2.8 ± 0.7 (2–6) days. Of the 56 patients, 46 had renal cell carcinoma, and 10 patients had benign histological findings. The surgical margins were positive in two patients. One of those patients with positive surgical margins had a 1.5 cm tumor size. PADUA and RENAL nephrometry scores of this patient were 6 and 4 respectively. The tumor was located on the anterior aspect and lower pole of the kidney. The other patient had a 4 cm tumor size, and 7 and 5 PADUA and RENAL nephrometry scores respectively. The tumor was located anteriorly at the upper pole of the kidney. The mean radiological tumor size of all patients was 3.1 ± 1.1 cm. Patient demographics and tumor characteristics are given in Table 1, and perioperative and postoperative outcomes are given in Table 2. There was no evidence of cancer recurrence in the patients at the follow-up.

The mean serum creatinine levels were 0.86 ± 0.5, 0.92 ± 0.6, and 0.88 ± 0.7 mg/dL in the preoperative, immediate postoperative, and postoperative 6^th^ month periods, respectively. The eGFR values were 91.8 ± 22.7, 85.6 ± 23.4, and 90.5 ± 16.6 in the preoperative, immediate postoperative, and postoperative 6^th^ month periods, respectively. There were no statistically significant differences, between preoperative eGFR and serum creatinine levels, compared with those of immediate postoperative and postoperative 6^th^ month periods (p > 0.05). The comparison of renal functions of patients, between the preoperative, immediate postoperative, and postoperative 6^th^ month visits is given in Table 3.

## 4. Discussion

Although the treatment of kidney tumors is still RN, PN is preferred widely in most of the stage T1 renal masses [2]. The reports of RPN in the literature show that it is a feasible method with good oncological, and functional outcomes [10–16]. Therefore, RPN is a practical minimally invasive procedure compared with laparoscopic surgery, which necessitates improved instrumentation capability.

Renal ischemia decreases the GFR and causes acute kidney injury in several ways including, tubule obstruction, persistent vasoconstriction, and reperfusion injury [17]. The warm ischemia time causing ischemic injury in the kidney is still a challenging issue of PN, eventually, any amount of damage might occur after renal arterial clamping. Robotic surgery provides several advantages, enabling quicker tissue dissection, reconstruction, and intracorporeal suturing, and facilitating the duration of surgery, that may result in better functional outcomes. Furthermore, zero ischemia RPN prevents ischemic renal injury and has a favorable effect on postoperative functional outcomes. During conventional RPN procedures, the renal artery is clamped, and total renal arterial flow is cut off temporarily. Therefore, it is expected that postoperative kidney function may deteriorate in patients with accompanying kidney diseases.

Several techniques were described to reduce ischemic damage during PN, including super selective versus main renal arterial control, and the use of near-infrared fluorescence (NIRF) imaging, controlling the arteries that supply the tumor. In the latter procedure, NIRF imaging verifies the selective renal ischemia, after indocyanine green dye administration [18]. In a prospective study including 36 patients, the feasibility and effectiveness of robotic partial nephrectomy performed with segmental clamping of tumor-feeding arteries were evaluated [19]. The method was successful in 34 patients, and the authors considered it as a reliable and effective surgical method during RPN. Alternatively, early unclamping can also be done following completing excision of the mass and internal renorraphy in order to decrease the duration of complete renal ischemia. Consequently, all of these techniques were suggested as safe alternatives to conventional RPN that eliminate global renal ischemia. In 2011, Gill et al. presented their initial experience in fifteen patients, undergoing zero-ischemic laparoscopic or robotic PN with encouraging oncological and functional results [20]. Since then, many reports about zero ischemia PN have been published. Due to our recently published study including a limited number of patients, zero ischemia RPN was found to be superior in preserving renal function in the short-term follow-up, compared to on clamp approach suggesting a functional advantage of the zero ischemia technique [21]. However, in this current study conducted with a larger number of patients, no statistically significant changes were revealed in terms of functional outcomes in the short-term and postoperative 6^th^ month visits. A recently published prospective randomized trial analyzed the perioperative outcomes, and postoperative renal function of 80 patients undergoing RPN with off-clamp and on-clamp techniques [22]. The renal function was assessed by estimated glomerular filtration rate eGFR and renal scintigraphy preoperatively and at postoperative 3^rd^ month. In this prospective study, similar perioperative outcomes and renal functions were obtained in both groups. The authors suggested that both techniques might be safely employed depending on the surgeons’ preference and patient related factors such as baseline renal insufficiency, multiple masses, or solitary kidney. Despite the controversial results in the literature, the influence of renal function on oncological outcomes was supported in a recent multiinstitutional study [23]. In this retrospective study, data of 3457 patients who underwent radical (39%) or partial nephrectomy (61%) for cT1–2 renal tumors were evaluated. As a result, the authors found a correlation between renal function and cancer specific mortality that should be taken into account in patients undergoing surgery for renal cancer.

The amount of estimated blood loss might be higher in zero ischemia RPN procedures, however, favorable outcomes with acceptable complication rates could also be achieved. Undoubtly, the complication rates might lessen with growing experience of zero ischemia RPN. However, to avoid the risk of major hemorrhage, a bulldog clamp may be kept close to the surgical field to clamp the renal artery in case of excessive bleeding. There are also several studies favoring direct tumor focusing without hilar vascular dissection. In a retrospective study, the results of off-clamp laparoscopic PN without dissecting and controlling the renal hilus was evaluated in 58 renal units with low tumoral complexity. Good oncologic and surgical results were obtained with acceptable complication rates. Consequently, the method was suggested as a preferable option for small sized, low-complex renal tumors [24].

In a recently published study [25], the outcomes of zero ischemia robotic and laparoscopic PN were evaluated for kidney tumors larger than 4 cm. A total number of 121 (70 laparoscopic and 51 robotic) patients underwent PN with controlled hypotension. The patients are divided into two groups according to the tumor size. Operative data, complications, serum creatinine, eGFRs and effective renal plasma flow were compared. Significant differences were detected in mean intraoperative hemorrhage (168 mL vs. 205 mL) and postoperative complications (6.4% vs. 18.6%). The authors concluded that, laparoscopic and robotic PN with controlled hypotension is applicable in kidney tumors larger than 4 cm with increased intraoperative hemorrhage and postoperative complications. Additionally, the authors also mentioned that, the technique prevents renal hilar clamping, which contributes to better functional outcomes.

Another study presented the initial outcomes of zero-ischemia RPN for complex tumors located at the renal pedicle [26]. The patients had a median tumor size of 4.1 cm, and the technique was accomplised in all patients with no intraoperative complications. On pathology of the tumor specimens, the surgical margins were clear. Besides, there were no statistically significant changes in serum creatinine and eGFR at discharge. In our series, there were 2 patients with positive surgical margins and the tumor sizes were 1.5 cm and 4 cm in those patients.

In a multiinstitutional study, perioperative and functional outcomes were compared in patients undergoing on-clamp and zero-ischemia RPN [27]. In the zero ischemia group, the operation time and eGFR decrease was less, but more hemorrhage was detected. The authors concluded that zero-ischemia RPN was safe and feasible in patients that have small kidney tumors.

R.E.N.A.L. nephrometry and PADUA scoring systems have been developed to describe the anatomic features of renal tumors in CT and MRI, and to predict surgical complications and oncological outcomes. The tumors are classified as low (4–6) or medium-high (7–12) in R.E.N.A.L. nephrometry classification. Similarly, in PADUA classification, tumors get scores between 6 and 14. Therefore, peri- and postoperative complication risks increase with higher scores. In our study, low complication rates were seen, consistently with low R.E.N.A.L. nephrometry and PADUA scores.

In our series, pedicle dissection was not performed in two cases with completely exophytic masses. The masses were located at the lower pole of the kidney and had 1.5 and 3.2 cm diameter. The procedures were completed successfully in 25 and 40 min, respectively, without any significant bleeding. Allthough encircling the renal artery and vein with vascular tapes is strongly recommended before resection of the mass, in limited cases, tumor resection may be performed safely without vascular dissection.

In our study, intraoperative endoscopic ultrasound was used in one patient who had a completely endophytic 3 cm lower pole mass. Intraoperative ultrasound may be required during RPN to show the tumor depth before starting tumor excision. In this patient, the histopathologic evaluation revealed clear cell RCC with a negative surgical margin.

Decision to perform zero ischemia RPN on the patients included in our series was related to surgeon’s decision. Our series included mostly cT1a renal masses less than 4 cm in size that were not complex masses and that might explain favorable outcomes obtained in our results. Nevertheless, we had 3 patients with tumor sizes of larger than 4 cm (5.5, 6.6, and 7.0 cm) suggesting that zero ischemia RPN might also be feasible in selected larger renal masses. In our series, perioperative complication was seen in one patient due to major hemorrhage which required blood transfusion and anjiographic intervention. Additionally, another patient was hospitalized because of prolonged subileus and received pharmacological treatment. Tumor sizes, CCI, R.E.N.A.L. nephrometry, and PADUA scores were 7 cm, 3, 8, and 8 in the first patient and were 3 cm, 3, 5, and 7 in the second patient.

According to post hoc power analysis, when the effect of surgery on eGFR is examined, it can be said that the results of the study are sufficient to generalize, but when we examine the effect of surgery on creatinine, the results of the study should be supported by similar studies with large samples. In this study, we present the favorable outcomes of RAPN with minor complications in 56 patients. Additionally, if the patients undergoing zero ischemia technique were compared with a group that underwent on-clamp RAPN, we might have a better knowledge of oncologic and functional outcomes. Another limitation of the study is; the procedures were performed at three institutions by four console surgeons. Despite the wide experience of the console surgeons, the large number of institutions and surgeons makes it harder to standardize the results. However, the most important aspect that makes the study strong, is that the results revealed are based on a high level robotic experience.

## 5. Conclusions

In summary, our results confirmed that, zero ischemia RPN is a safe and feasible method for the treatment of T1 renal tumors in accordance with the literature. It has acceptable oncological and functional outcomes in small renal masses and even in selected larger renal masses. Despite the growing number of studies on on-clamp and zero ischemia RPN, comparative prospective studies evaluating the impact of both methods on renal functions and including large patient numbers with long term follow-up are still lacking,

## Figures and Tables

**Table 1:** Tumor characteristics.

Variable	Value
Sex: Male/female	41/15
Number of patients	56
Mean (SD, range):	
Age	52.2 ± 8.1 (27–75)
Body mass index (BMI)	27.8 ± 3.5 (22.6–36.3)
Radiological tumor size (cm)	3.1 ± 1.1 (1–7)
Pathological tumor size (cm)	2.8 ± 1.4 (1–7.5)
PADUA	7.3 ± 1.0 (6–9)
R.E.N.A.L. nephrometry	6.1 ± 1.3 (4–9)
Histopathological results	
Renal cell cancer	46 (33 clear cell, 9 chromophobe cell, and 4 papillary cell)
Oncocytoma	6
Other (angiomyolipoma, fibroadipose tissue, papillary epithelial hyperplasia)	4
Fuhrman grades	Grade I (4 patients) Grade II in (34 patients) Grade III (8 patients)

**Table 2:** Perioperative and postoperative outcomes of the patients.

Variable	Value
Console time (min)	108.4 ± 18.2
Estimated blood loss (mL)	166.2 ± 124.7
Length of hospital stay (days)	2.8 ± 0.7 (2–6)
Follow up (months)	33.6 ± 12.3 (3–76)
Intraoperative complication	0
Perioperative complication	2 (Clavien 1 and Clavien 3)

**Table 3:** Comparison of renal functions between the preoperative, immediate postoperative, and postoperative 6^th^ month visits.

Pre-operative creatinine	Immediate ostoperative creatinine	p	Preoperative creatinine	Postoperative 6^th^ month creatinine	p
0.86 ± 0.5	0.92 ± 0.6	0.12	0.86 ± 0.5	0.88 ± 0.7	0.17
**Preoperative eGFR**	**Immediate postoperative eGFR**	**p**	**Pre operative eGFR**	**Post-operative 6** **^th^** ** month eGFR**	**p**
91.8 ± 22.7	85.6 ± 23.4	0.33	91.8 ± 22.7	90.5 ± 16.6	0.41

Wilcoxon’s signed- rank test, statistical significance p = 0.05.
